# A low-cost open-source imaging platform reveals spatiotemporal insight into leaf elongation and movement

**DOI:** 10.1093/plphys/kiae097

**Published:** 2024-02-24

**Authors:** Lisa Oskam, Basten L Snoek, Chrysoula K Pantazopoulou, Hans van Veen, Sanne E A Matton, Rens Dijkhuizen, Ronald Pierik

**Affiliations:** Plant-Environment Signaling, Department of Biology, Utrecht University, Utrecht 3584 CH, The Netherlands; Theoretical Biology and Bioinformatics, Department of Biology, Utrecht University, Utrecht 3584 CH, The Netherlands; Plant-Environment Signaling, Department of Biology, Utrecht University, Utrecht 3584 CH, The Netherlands; Plant-Environment Signaling, Department of Biology, Utrecht University, Utrecht 3584 CH, The Netherlands; Laboratory of Molecular Biology, Wageningen University and Research, Wageningen 6700 AA, The Netherlands; Plant-Environment Signaling, Department of Biology, Utrecht University, Utrecht 3584 CH, The Netherlands; Plant-Environment Signaling, Department of Biology, Utrecht University, Utrecht 3584 CH, The Netherlands; Laboratory of Molecular Biology, Wageningen University and Research, Wageningen 6700 AA, The Netherlands

## Abstract

Plant organs move throughout the diurnal cycle, changing leaf and petiole positions to balance light capture, leaf temperature, and water loss under dynamic environmental conditions. Upward movement of the petiole, called hyponasty, is one of several traits of the shade avoidance syndrome (SAS). SAS traits are elicited upon perception of vegetation shade signals such as far-red light (FR) and improve light capture in dense vegetation. Monitoring plant movement at a high temporal resolution allows studying functionality and molecular regulation of hyponasty. However, high temporal resolution imaging solutions are often very expensive, making this unavailable to many researchers. Here, we present a modular and low-cost imaging setup, based on small Raspberry Pi computers that can track leaf movements and elongation growth with high temporal resolution. We also developed an open-source, semiautomated image analysis pipeline. Using this setup, we followed responses to FR enrichment, light intensity, and their interactions. Tracking both elongation and the angle of the petiole, lamina, and entire leaf in Arabidopsis (*Arabidopsis thaliana*) revealed insight into R:FR sensitivities of leaf growth and movement dynamics and the interactions of R:FR with background light intensity. The detailed imaging options of this system allowed us to identify spatially separate bending points for petiole and lamina positioning of the leaf.

## Teaser

A modular and low-cost phenotyping setup with an open-source image analysis pipeline shows the importance of spatial and temporal resolution in understanding differential leaf elongation.

## Introduction

Plant life is centered around sunlight, and ensuring access to this vital energy resource is crucial for plants to power photosynthesis. This is particularly challenging in dense vegetation, both in natural and agricultural fields, where plants compete for access to this resource because they mutually shade each other (Casal 2013; Huber et al. 2021). Upward leaf movement, called hyponasty, elevates the photosynthetically active leaf tips toward higher zones of the vegetation where light availability is improved compared with lower regions. Upward leaf movement is part of the shade avoidance syndrome (SAS) that promotes light capture in dense stands and furthermore includes accelerated elongation growth of stems and petioles, apical dominance, and early flowering (Casal 2013; Pierik and Ballaré 2021). Interestingly, leaf movements do not just occur in response to competition but are in fact regulated by a wide variety of cues and environmental conditions. This repositioning can be downward (epinasty) or upward (hyponasty). Adaptive relocation of leaves and petioles for example allows plants to align their leaves with the sun to facilitate light capture or move them away to prevent photodamage (Fu and Ehleringer 1991; Pérez-Llorca et al. 2019). Leaf angle changes to avert photodamage are further influenced by drought (Pastenes et al. 2005). Upward movement of rosette leaves is also a coping mechanism for high temperature since it facilitates cooling (Koini et al. 2009; Park et al. 2019) and for flooding-escape as it may enable aerial contact (Cox et al. 2003; Lee et al. 2011). Inhibiting leaf movements by physically constraining the leaves was found to negatively affect leaf growth (Walter et al. 2002; Cox et al. 2003). Circadian leaf movements allow diurnal growth and may facilitate overtopping of leaves of neighboring plants, thus aiding in competition (Woodley of Menie et al. 2019). Overtopping of neighbors is also directly enabled by neighbor-induced hyponasty in response to light cues from neighbors (Bongers et al. 2018; Huber et al. 2021).

Neighbor proximity is perceived by plants through changes in the red (R) to far-red (FR) ratio and blue light levels (Ballaré et al. 1990; de Wit et al. 2016). A low R:FR ratio results from selective FR light reflection by neighbors, which can be perceived locally at the leaf tip by phytochrome B (phyB), subsequently leading to derepression of PHYTOCHROME INTERACTING FACTORs (PIF) 4, 5, and 7 and resulting in a hyponastic response of 1 specific leaf (Pantazopoulou et al. 2017). Neighbor proximity and shade results in upward movement of the petiole and leaf (Millenaar et al. 2009; de Wit et al. 2012; Michaud et al. 2017; Pantazopoulou et al. 2017). Upward movement of the petiole is caused by differential elongation between the abaxial and adaxial side (Polko et al. 2012; Küpers et al. 2023). These light-regulated movements are however often studied at relatively low temporal resolution (Pantazopoulou et al. 2017) or very young plants (Michaud et al. 2017), whereas it is known that (subtle) differences in hyponastic response can influence adult plant fitness at the canopy level (Bongers et al. 2018; Woodley of Menie et al. 2019; Pantazopoulou et al. 2021). Altering leaf position can be achieved by adjusting the angle via differential growth rates of the lamina, petiole, or both parts of the leaf simultaneously. The parts of the leaf that can elicit the biggest change in angle are the proximal petiole region (Küpers et al. 2023) and potentially the lamina–petiole junction. Together, this allows for potential independent petiole and lamina angle adaptations for optimal light capture during competition, but such details have not been studied. FR treatments are typically done through discrete FR additions providing strong R:FR ratio reductions, whereas in reality, R:FR ratios can fluctuate very broadly. The quantitative relationship between the intensity of the FR signal and the leaf response has not been described. Furthermore, R:FR light variations are often accompanied by fluctuations in light intensity, adding an additional layer of complexity to the regulation of leaf movement that is not well understood. Understanding and further elucidating the mechanisms regulating the hyponastic response requires a high temporal resolution of petiole, lamina, and whole leaf movement, as well as a detailed look at the tissues, and their plasticity, that contributes to overall leaf positioning in a canopy.

Measuring petiole and lamina movement and elongation through day and night with a high temporal resolution would aid in further elucidating molecular regulation of leaf movement. Measuring leaf movement was previously achieved with conventional RGB time-lapse imaging (Cox et al. 2003; Millenaar et al. 2005; Rehman et al. 2020). A major disadvantage however is that RGB time-lapse imaging depends on white light (WL), which interferes with photoreceptor activity, and nighttime imaging in light will likely affect leaf movements. Using infrared (IR) lighting and imaging would help solve this potential problem. In some setups, leaf lamina and petiole movement are not directly measured but derived from images from above rather than the side, estimating the leaf angle from variations in projected leaf length (Bours et al. 2012; Rehman et al. 2020). This form of imaging makes it difficult to distinguish whether leaf movement is caused by petiole or lamina movement and is a derived measure from interpolated growth rates which vary greatly throughout the day. Other high-throughput phenotyping solutions are developed and work well to quantitate leaf movements in great detail. For example, attachable digital inertial measurement unit (IMU) sensors were deployed to measure leaf movement of larger crop species, such as banana, tomato, and bell pepper (Geldhof et al. 2021). However, IMU sensors are still too large for use on small leaves such as those of Arabidopsis. Alternatively, 3D laser scanner-based leaf angle imaging methods combined with a robotic stage are successfully used in leaf angle quantifications through time (Dornbusch et al. 2012; Michaud et al. 2017). However, such setups are major financial investments and rely on sophisticated downstream data analytics pipelines, making these solutions relatively inaccessible to many research environments around the globe. Another potential drawback of such setups is the relatively modest temporal resolution: laser scanners can cause heat damage prohibiting very frequent imaging (Dornbusch et al. 2012), and conveyer belt-based automated systems for measuring multiple individuals leave a relatively long time between repeated measurements on the same individual (Apelt et al. 2015). Thus, taken together, the available methods are often subject to setup-specific limitations or are too costly to be used by many research groups. There is, therefore, a need for an open-source, low-cost solution, such as those enabled by minicomputer-based imaging (Tovar et al. 2018).

Here, we present a low-cost imaging system with high temporal resolution to accurately follow petiole and lamina movement and growth through time, both during day and night conditions using IR light-emitting diodes (LEDs). We also developed an open-source semiautomated image analysis pipeline to analyze petiole, lamina, and whole leaf angles and elongation in Arabidopsis. We deployed our setup to explore how different strengths of R:FR ratio reduction regulate petiole and lamina elongation and hyponasty kinetics. Such FR dose–response data were not previously available and revealed that FR responsiveness scales differently between elongation and hyponastic movement. Additionally, we studied if whole plant FR (FRw) treatments elicit similar leaf movement and growth kinetics as does localized FR treatment to leaf tips and how these interact with background light intensity. Lastly, we studied the impact of canonical shade avoidance regulators PIF4, PIF5, and PIF7 on petiole and lamina angle regulation. These data together show the abilities of the setup and data pipeline and shed light on light control of hyponastic leaf movement, thus opening research avenues into molecular control of leaf movement.

## Results

**Figure 1.**
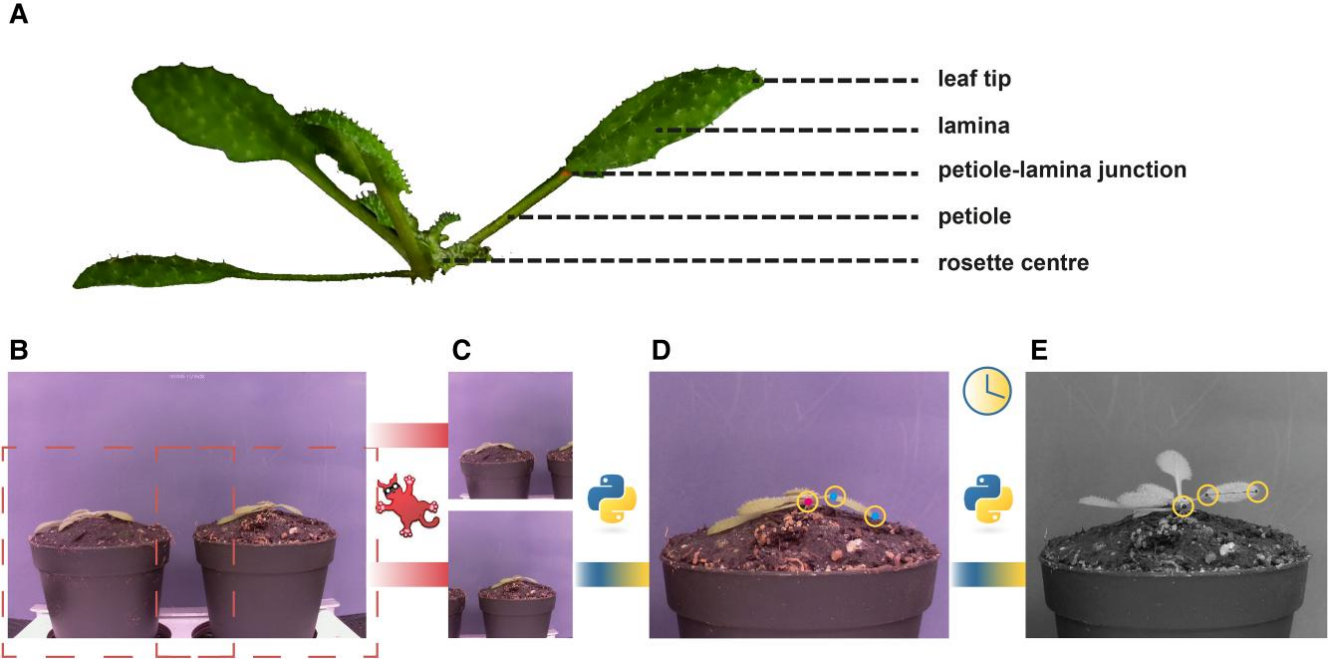
Workflow of the semiautomated imaging pipeline. Picture of an hyponastic Arabidopsis plant, with different parts of the leaf indicated **(A)**. Example picture taken by RPi camera; **B)** is cropped using IrfanView to create 2 separate images each containing a single plant **(C)**. With a Python script, 3 locations are user-selected, as indicated by the yellow circles **(D)**. Petiole–lamina junction and leaf tip are selected in the first image (cyan dots). Rosette center is selected on the first and last image (magenta dot). The script will locate these points in all subsequent images **(E)**.

**Figure 2.**
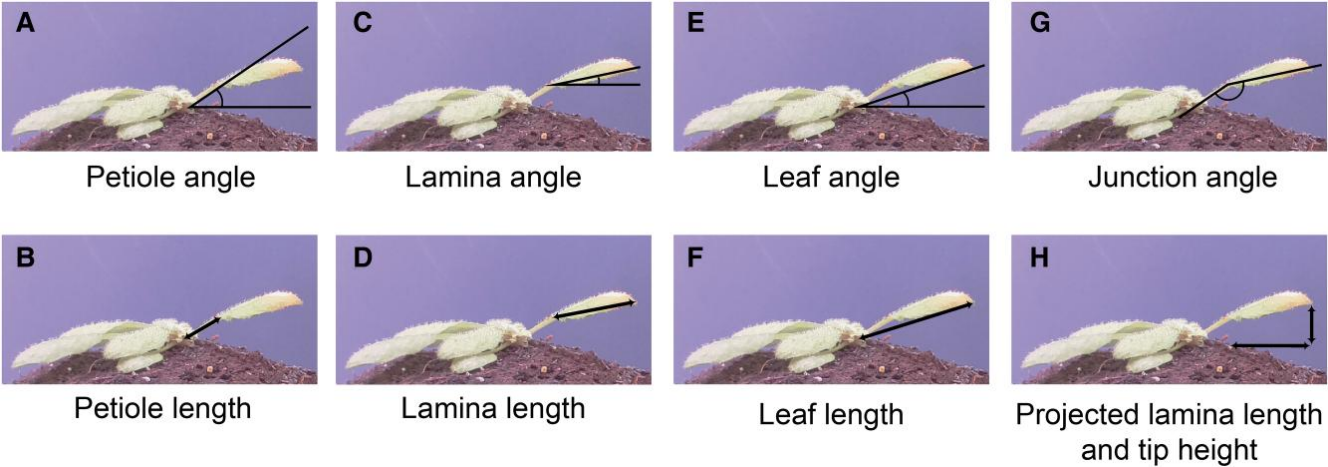
Measurements derived from images taken by the imaging setup. From rosette heart to petiole–lamina junction: petiole angle **(A)** and petiole length **(B)**. From petiole–lamina junction to lamina tip: lamina angle **(C)** and lamina length **(D)**. From rosette center to lamina tip: leaf angle **(E)** and leaf length **(F)**. Lamina angle relative to the petiole angle: junction angle **(G)**. Lamina length from petiole–lamina junction to leaf tip based solely on the *x* coordinates and lamina tip height based on *y* coordinates from the lamina tip and the rosette heart: projected lamina length and tip height **(H)**.

In response to supplemental FR light that signals neighbor proximity, Arabidopsis plants typically move their leaves upward (Michaud et al. 2017; Pantazopoulou et al. 2017). The response is considered to occur primarily from the petiole base and results from differential elongation between the abaxial versus adaxial side of the proximal petiole region (Küpers et al. 2023). Nevertheless, the angle of the lamina specifically could also change, and the differential elongation for specific lamina movements would then take place in the lamina–petiole junction. To accurately track differential elongation through time in combination with measuring elongation growth, it is essential to monitor the lamina and the petiole separately. Using the images taken in our setup and the downstream semiautomated image analysis pipeline (Fig. 1), petiole, lamina, leaf, and junction angles can be measured, together with the lengths of the petiole, lamina, and leaf (Fig. 2). The leaf tip and lamina–petiole junction are based on user-defined locations in the first image. These are then followed in all subsequent images through channel and spatial reliability tracking (CRST)-based automated tracking of the *x* and *y* coordinates of the moving lamina tip and the lamina–petiole junction using OpenCV (Bradski 2000; Lukežič et al. 2018; Xu et al. 2019). The information inside a bounding box surrounding the initial user-defined locations is combined with information outside the bounding box, using online machine learning to create a discriminative correlation filter (Lukežič et al. 2018; Xu et al. 2019). This approach allows the program to recognize the petiole–lamina junction and the leaf tip in the subsequent images. These changing positions are recorded against the user-defined *x* and *y* coordinates of the petiole origin at the rosette center (Fig. 1, C and D) in order to do downstream calculations of lengths and angles. Petiole length and angle are calculated from rosette center and petiole–lamina junction coordinates, lamina length and angle from petiole–lamina junction and lamina tip coordinates, and leaf angle and length from rosette center and leaf tip coordinates. From these measurements, several derived components can be quantified, such as the junction angle, the leaf tip elevation, and the projected lamina length (Fig. 2H). The leaf tip elevation and projected lamina length combine the angle with the elongation, showing the total absolute vertical and horizontal displacement of the leaf tip, which helps interpret the plant's competitiveness.

### Subtle differences in timing exist between petiole and lamina movement response to FR light

**Figure 3.**
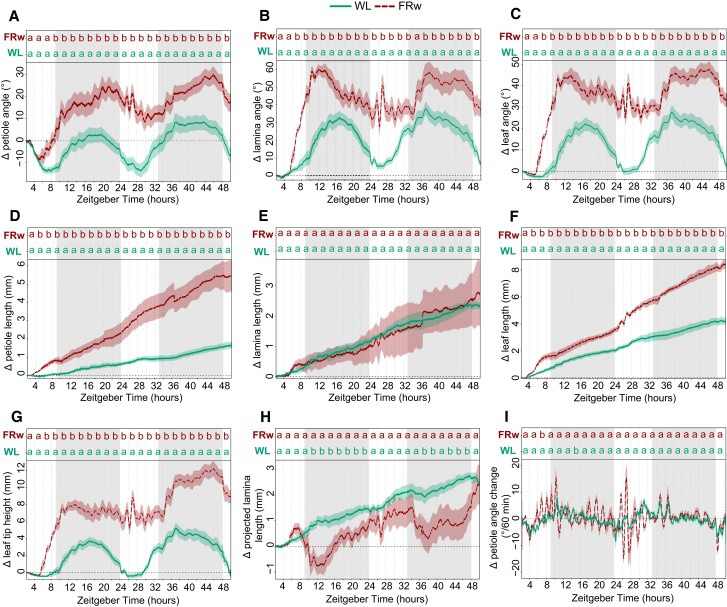
Plant response kinetics to WL or WL with supplemental FR light on the entire plants (FRw). Relative angle change of petiole **(A)**, lamina **(B)**, or total leaf **(C)**. Relative length change of petiole **(D)**, lamina **(E)**, or total leaf **(F)**. Relative leaf tip elevation **(G)**, relative change in projected lamina length **(H)**, and speed of petiole angle adjustment over 60 min periods **(I)**. Shaded areas indicate dark period without light or FR exposure. Plants were followed for 48 h and treated with FRw, R:FR = 0.1, or control WL. Treatment start time at ZT = 2, WL *n* = 7, FRw *n* = 6. PAR = 140 *µ*mol m^−2^ s^−1^ for both treatments. Lines represent mean ± Sem. Letters indicate *P* < 0.05, calculated per every 2 h using 1-way ANOVA and Tukey post hoc test. Gray areas indicate night without light or FR exposure.

Using the imaging setup and analysis pipeline, we explored leaf responses of plants that were placed for 48 h in either a control WL (R:FR = 1.5) or WL supplemented with uniform FRw light (R:FR = 0.1) (Fig. 3). Images were acquired every minute and processed for quantitative data extraction. Control WL plants already move their petioles and laminas in a diurnal pattern (Fig. 3, A and B), as previously reported (Michaud et al. 2017; Woodley of Menie et al. 2019). Lamina and petiole patterns are alike in control conditions, but this pattern in movement dynamics between petiole and lamina changes when exposed to a low R:FR ratio, which had not been reported before. In FRw, the lamina and leaf angle change peak at the beginning of the night around ZT 12, whereas the petiole angle change reaches the maximum at the end of the night around ZT 22 (Fig. 3, A to C). The junction angle in FRw only differs from WL leading up toward the first night and during the beginning of the first night, starting from ZT 6 until approximately ZT 15 (Supplementary Fig. S1A). FRw-treated plants display a stronger oscillating pattern than WL, indicating rapid angle adjustments and changes in elongation pace (Fig. 3I and Supplementary Fig. S1B). Oscillation occurs first during the first night, with even more pronounced peaks during the second day between ZT 25 and ZT 28.

Petiole and lamina elongation show different FR responses: FRw strongly promotes petiole elongation, whereas lamina length is not affected by FRw (Fig. 3, D to F). Petiole elongation seems to show a difference between FRw and WL slightly earlier than the hyponastic response: around ZT 4 for petiole elongation versus ZT 6 for petiole angle. Leaf tip elevation moves up and lowers again over the course of 24 h in WL (Fig. 3G),but further rises over the second 24 h, driven by both endogenous petiole and lamina movement and petiole elongation. These leaf tip elevations are strongly enlarged in the presence of supplemental FR light. Projected lamina elongation is mostly affected during the night for FRw-treated plants (Fig. 3H), whereas during the day, this is not different from WL.

Considering the differences in response patterns between the petiole and lamina when exposed to a low R:FR ratio, tracking these organs separately is necessary to accurately monitor plant responses and inform follow-up studies into the molecular regulation of these events that need to be conducted on the appropriate tissues.

### FRw dosage determines the strength of leaf responses

**Figure 4.**
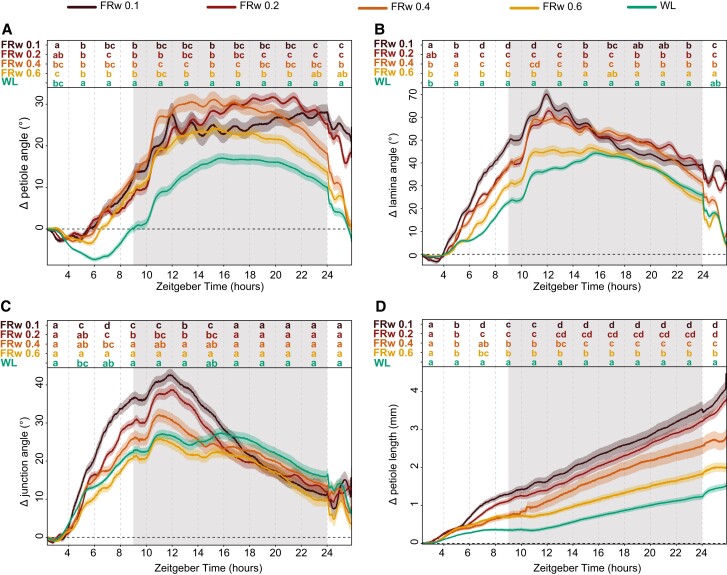
FRw intensity influences the strength of leaf responses differently between organs. Relative angle change of petiole **(A)**, lamina **(B)**, and petiole–lamina junction **(C)** and relative length change of petiole **(D)**. Treatments consisted of control (WL) and 4 FRw doses with R:FR 0.1, 0.2, 0.4, and 0.6 (FRw 0.1, FRw 0.2, FRw 0.4, and FRw 0.6), with 140 *µ*mol m^−2^ s^−1^ PAR for all treatments. Measured for 24 h with treatment start time at ZT 2, WL *n* = 69, FRw R:FR 0.1 *n* = 22, FRw R:FR 0.2 *n* = 31, FRw R:FR 0.4 *n* = 28, and FRw R:FR 0.6 *n* = 30. Lines represent mean ± Sem. Letters indicate *P* < 0.05, calculated per every 2 h using 2-way ANOVA and Tukey post hoc test. Shaded areas indicate night without light or FR exposure.

Next, we aimed to investigate the relationship between FR light intensity and leaf responses. We added different fluence rates of FR and maintained a standard background photosynthetically active radiation (PAR: 400 to 700 nm waveband), creating stronger reductions of R:FR ratios with increasing FR intensities. Petiole hyponastic responses are congruent for all 4 FRw fluence rates during the first hours of the response but diverge during the night period into 2 groups (Fig. 4A) that all have higher petiole angles than the WL control. However, in the second light period, the 2 higher R:FR ratios (i.e. mild FR enrichments) converge with WL 24 h posttreatment start time, whereas R:FR = 0.1 and 0.2 (i.e. strong FR enrichments) maintain their elevated petiole angles. The lamina angle change during the first day and the beginning of the night is increasing proportionally with increasing FR fluence rates, but after 24 h of treatment, the groups have diverged similarly the petiole hyponastic response (Fig. 4B). Both petiole and lamina angle display relatively rapid synchronous oscillations during the night period, with more pronounced oscillations for R:FR = 0.1 and 0.2 (Fig. 4, A and B), an observation that has not previously been described. Junction angle change shows a similar pattern as the lamina angle, except that the mildest FR treatment (R:FR = 0.6) is barely different from WL control (Fig. 4C). Petiole elongation shows an increasing response to increasing FR fluence rates (Fig. 4D). Looking more closely at the elongation pattern in the first hours, all 4 different FRw treatments group together from approximately ZT 4 to ZT 6. Around ZT 8, the petiole elongation of 2 lowest R:FR ratio groups, FRw 0.1 and 0.2, is significantly higher than that of the 2 slightly higher R:FR ratio treatments of 0.4 and 0.6. Around ZT 15, the R:FR = 0.4 group show significantly more petiole elongation from the R:FR = 0.6 group, resulting in the gradual increase of petiole length after 24 h with lowering of R:FR that was previously reported (Bongers et al. 2018).

Increasing supplemental FR fluence rates lead to increasingly strong responses of petiole and lamina growth and movement. However, for the different organs and responses, there are subtle differences: whereas petiole length increase straightforwardly corresponds with FR intensity, petiole angle increase is rather similar between all FR fluence rates initially, but the longer-term maintenance of an elevated angle requires a rather high fluence rate of FR light. For the lamina, the initial angle increase response is more gradual at the onset compared with the petiole angle but needs a similarly high fluence rate of FR light to maintain the elevated lamina angle after 24 h. Together, these results highlight the importance of having both a high temporal resolution and the opportunity to track lamina and petiole separately when assessing the hyponastic response, in order to understand both timing and tissue localization of leaf positioning.

### Light intensity affects petiole hyponastic response to FRw but not local FR

**Figure 5.**
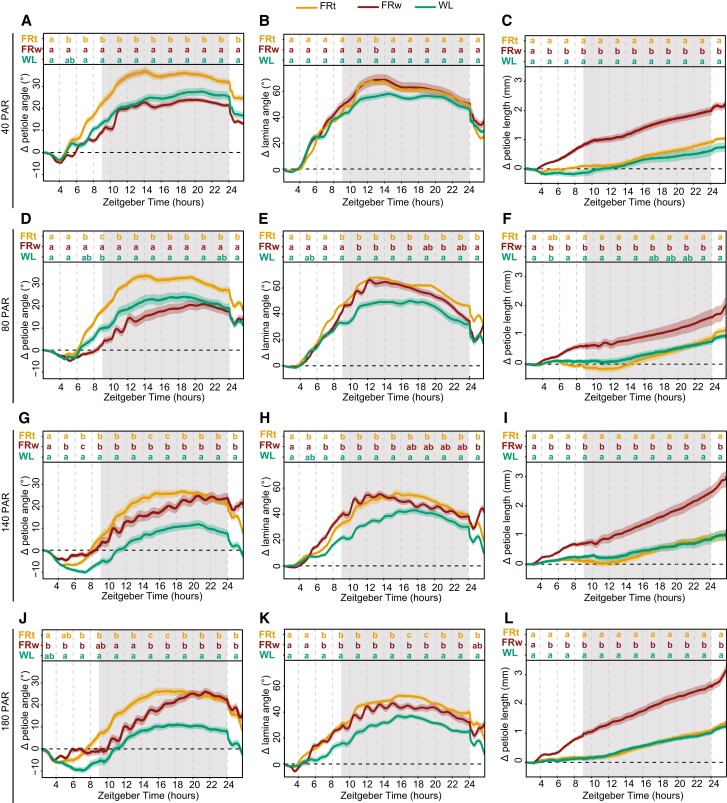
Interaction between background light intensity and local or FRw treatments. Relative angles of petiole and lamina and relative petiole elongation with a background light intensity of 40 **(A** to **C)**, 80 **(D** to **F)**, 140 **(G** to **I)**, and 180 *µ*mol m^−2^ s^−1^ PAR **(J** to **L)**. Treatments consisted of supplemental local FRt, FRw, and WL control. 40 PAR WL *n* = 12, FRt *n* = 24, FRw *n* = 24; 80 PAR WL *n* = 11, FRt *n* = 22, FRw *n* = 27; 140 PAR WL *n* = 16, FRt *n* = 19, FRw *n* = 25; and 180 PAR WL *n* = 23, FRt *n* = 22, FRw *n* = 23. Measured for 24 h with treatment start time at ZT 2. Lines represent mean ± Sem. Letters indicate *P* < 0.05, calculated per every 2 h using 2-way ANOVA and Tukey post hoc test using the sample sizes indicated above. Shaded areas indicate night without light or FR exposure.

Dense canopies not only reflect FR light but also have reduced light intensities in the lower zones. As PAR and R:FR ratio are not homogenously distributed throughout the canopy, it is important to understand if and how responses to low R:FR are affected by PAR. We therefore moved our adult plants from control conditions of 140 *μ*mol m^−2^ s^−1^ PAR WL to either 40, 80, 140, or 180 *μ*mol m^−2^ s^−1^ PAR. This was combined with supplemental FRw with R:FR = 0.1, where the FR fluence rate was adjusted for every PAR condition such that the FR treatment always reached R:FR = 0.1, irrespective of the PAR combination treatment. We also included a second type of FR treatment: a spotlight FR irradiation to only the tip of the tracked leaf (FRt), leading to a highly localized drop of the R:FR ratio to 0.1 (Fig. 5). Local FR treatment on the leaf tip alone is sufficient to elicit a hyponastic response in the petiole and lamina of a magnitude similar to, or even higher than, FRw (Supplementary Fig. S2, A and B; Küpers et al. 2023; Michaud et al. 2017; Pantazopoulou et al. 2017). Plants moved to lower PAR than the control condition and increase petiole angles without additional FR required (Fig. 5, A, D, and G), a well-established response to low light (Millenaar et al. 2009). Surprisingly, at light intensities below 140 PAR, only FR treatment locally on the leaf tip elicits a hyponastic petiole response additional to the increase seen in the reduced WL fluence rate conditions (Fig. 5, A, D, and G), and FRw has no additional effect. Rapid oscillations however do persist, although reduced in frequency. As a separate control, we performed an experiment where we reduced the local FRt fluence rate with the same percentage as used to reduce PAR and found that this lower FRt fluence rate has the same effect as strong FRt (Supplementary Fig. S3, A and B). Lamina hyponastic response differs from petiole hyponastic response, since both FRt and FRw induced an additional angle movement in low PAR (Fig. 5, B, E, and H). Higher light intensity (PAR = 180) results in a delayed onset of FRw-induced petiole hyponasty compared with PAR = 140, combined with a stronger decline around ZT24 (Fig. 5, G and J), whereas FRt at PAR = 180 results in a similar response as in PAR = 140. Lamina angle changes in PAR = 180 remain similar to control conditions (PAR = 140) for all treatments (Fig. 5, H and K). Petiole elongation persists in FRw throughout all PAR conditions, although there is a trend toward milder responses at lower PAR (Fig. 5, C, F, I, and L).

These results indicate that there is an interaction between background light fluence rate and the location of low R:FR perception. Furthermore, the petiole and the lamina respond differently to these combinations of background light intensities and low R:FR treatments.

### Lamina–petiole junction can function as an additional pivot point to the petiole base during the hyponastic response

**Figure 6.**
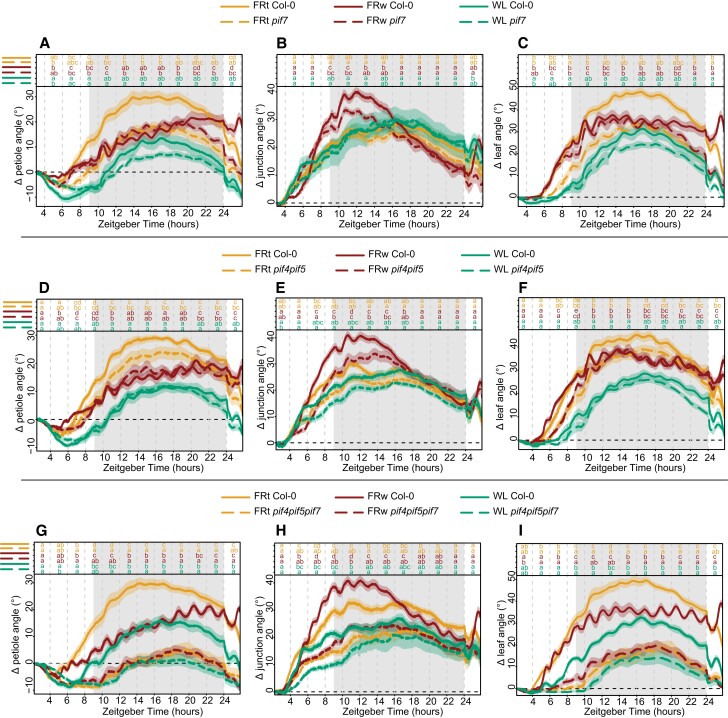
Core shade avoidance regulators PIF4, PIF5, and PIF7 regulate differential elongation in both petiole and lamina. Relative angles for petiole, petiole–lamina junction, and leaf in control (WL), local supplemental FRt, and FRw for mutants pif7 **(A** to **C)**, pif4pif5 **(D** to **F)**, and pif4pif5pif7 **(G** to **I)**. Col-0 WL *n* = 8, FRt *n* = 10, FRw *n* = 13, pif7 WL *n* = 7, FRt *n* = 11, FRw *n* = 17. Col-0 WL *n* = 7, FRt *n* = 10, FRw *n* = 17, pif4pif5 WL *n* = 7, FRt *n* = 10, FRw *n* = 15. Col-0 WL *n* = 9, FRt *n* = 11, FRw *n* = 15, pif4pif5pif7 WL *n* = 9, FRt *n* = 9, FRw *n* = 13. Measured for 24 h with treatment start time at ZT 2. Lines represent mean ± Sem of the mean. Letters indicate *P* < 0.05, calculated per every 2 h using 2-way ANOVA and Tukey post hoc test. Shaded areas indicate night without light or FR exposure.

The hyponastic response is initiated by phyB inactivation, derepressing the inhibition of PIF4, 5, and 7 (reviewed in Küpers et al. 2020). PIF4, 5, and 7 are required for the hyponastic response, with *pif7* showing a severely weakened response, *pif4pif5* a slightly less weakened response, and *pif4pif5pif7* a completely diminished response in previous studies on the leaf and petiole angle (Michaud et al. 2017; Pantazopoulou et al. 2017). To further validate the setup, we tested these 3 *pif* mutants in WL, FRw, and FRt (Fig. 6). As previously reported by Pantazopoulou et al. (2017), *pif7* petiole angle response is severely weakened for FRt (Fig. 6A). This pattern of reduced FRt responses is reproduced by the leaf angle, whereas the lamina-to-petiole junction angle is not different between *pif7* and Col-0 (Fig. 6, B and C). FRw was previously reported to still elicit a response in *pif7* in leaf angle (Michaud et al. 2017) but not in petiole angle after 24 h of treatment (Pantazopoulou et al. 2017). Here, we show that *pif7* initially does respond similarly to Col-0 but loses the increase in petiole angle and leaf angle at the end of the night unlike Col-0, resulting in no visible response after 24 h (Fig. 6, A and C). Both petiole and leaf angle response to FRt are mildly reduced in the *pif4pif5* mutant (Fig. 6, D to F). FRw treatment results in a *pif4pif5* petiole angle almost similar to Col-0 (Fig. 6D). However, *pif4pif5* leaf angle is slightly reduced during the first 8 h of FRw treatment, consistent with a reduced junction angle (Fig. 6, E and F). Triple mutant *pif4pif5pif7* does not respond to FRt or FRw treatment with hyponasty (Fig. 6, G to I), as previously reported (Michaud et al. 2017; Pantazopoulou et al. 2017). Additionally, this triple mutant displays a severely reduced, but not absent, diurnal movement of the leaf in WL (Fig. 6, G to I). The triple mutant does retain modest FRw-induced oscillations (Fig. 6, G and I). Together, these results corroborate previous findings for core shade avoidance regulators PIF4, PIF5, and PIF7 controlling leaf angle responses to supplemental FR. Additionally, we show here that the lamina–petiole junction can function as an additional pivot point in the leaf and is affected differently between *pif7*, *pif4pif5*, and *pif4pif5pif7.*

## Discussion

We developed a low-cost, modular phenotyping setup to record leaf angle and length dynamics with high temporal precision. Furthermore, we also developed an open-source image analysis pipeline to derive quantitative information from the acquired images. This allowed for refined analyses of leaf movement and elongation for the petiole and lamina separately. Using this setup and image analysis pipeline, the petiole and lamina responses to various FR treatments, as a proxy for neighbor signals, were explored. We showed that petiole and lamina angle responses varied not only between different R:FR treatment locations and ratios but also between the lamina and the petiole itself. These insights highlight both the potential of the setup and the complexity of plant movement.

### Spatial and temporal resolution from adjustable image acquisition and analysis pipeline

The current imaging setup is based on raspberry Pi (RPi) minicomputers; however, other companies have produced similar single-board computers. Any single computer board that can control a camera and store the images could suffice. Similarly, the camera itself can be replaced by versions with different focal lengths or other characteristics that are tailored to the intended use, allowing, for example, imaging larger plants from a greater distance. This further underscores the adaptability of this system. Furthermore, the setup is scalable with minimal costs, as the components are relatively inexpensive and widely available.

The subsequent image analysis is relatively straightforward and based on open-source software as well. As the points of interest are user-defined by clicking on the image, the script is adaptable as well to add or remove points of interest, for example, to allow different segmentations. The successful imaging under different light treatments during the photoperiod, together with the IR light imaging in the night period, indicates the robustness of the tracking abilities of the script. Some previously established imaging setups use images from above (Bours et al. 2012; Rehman et al. 2020), which requires integration of petiole and lamina angle changes with elongation growth into 1 leaf movement output. Other motion estimation programs are converting the different motions of separate plant organs such as cotyledons and emerging true leaves into 1 derived relative motion output (Greenham et al. 2015).

In the setup presented here, a single leaf is imaged from the side, and angles and growth of the different leaf components are measured using basic triangulation (Figs. 1 and 2). There are some drawbacks to the current analysis method. Firstly, petioles and laminas are not always truly a straight line. Any curvature of either the petiole or lamina is currently not considered. However, adding more intermediate tracing points to the petiole and lamina would greatly improve the resolution in shape detection and is a relatively straightforward elaboration of the image analysis scripts. Secondly, because the leaf must be parallel to the camera, only 1 leaf per plant at a time can be measured accurately. Even though local FR signaling is known not to cause a response in other leaves (Pantazopoulou et al. 2017), FRw certainly does affect several leaves simultaneously, and so do other environmental parameters such as elevated temperature (Koini et al. 2009).

Advantages of the presented setup on the other hand are the high spatial and temporal resolution. This allows for the separate analyses of petiole and lamina angles and elongation (Figs. 2 and 3) and eliminates the need for integration of these separate processes. Secondly, combined and derived measurements of these separate traits provide further insight into the potential benefits under high planting densities. Tip height for instance could be used as an indication for overtopping potential. Projected lamina length combines the angle with elongation to give an indication of the area that is covered by the lamina and could potentially be used as a proxy for its shading surface area potential. Thirdly, with a datapoint every minute, elongation rate and angle change can be measured in great detail (Fig. 3I, Supplementary Fig. S1), which can guide future studies into the molecular mechanisms regulating the different phases of these responses.

### Insights from enhanced temporal resolution

The high resolution of the setup presented here provides detailed response kinetics on the elongation and angle changes of both the lamina and the petiole when exposed to a range of different neighbor proximity mimicking signals (Figs. 3 to 5). Several separate studies have investigated FR light-regulated leaf movement but typically not with the high spatial and temporal resolution used here (Dornbusch et al. 2014; Michaud et al. 2017; Pantazopoulou et al. 2017; Bongers et al. 2018; Küpers et al. 2023). This comprehensive data set was therefore not previously available and increases the resolution and depth of existing knowledge. For example, petiole elongation has been previously found to correspond with FR intensity (Bongers et al. 2018). Our results not only corroborate this finding (Fig. 4D) but also extend it.

We now see that the initial petiole elongation responses for the different FRw intensity treatments are similar to one another and only diverge after several hours of treatment. This implies that the early initiation of supplemental FR-induced petiole elongation is much more sensitive to modest FR enrichment than is the longer-term acceleration and consolidation. This might indicate that different molecular regulators drive the early versus later response. Indeed, in very young Arabidopsis seedlings, it has been proposed that prolonged FR-induced elongation involves a rewiring of auxin signaling as compared with the early FR-induced auxin response (Pucciariello et al. 2018). Although it is unknown if similar mechanisms regulate petiole elongation responses to FR in adult plants, it does present an example of how different phases of shade avoidance responses can involve different modes of regulation and how time-resolved information is key to resolving these mechanisms.

The importance of capturing the kinetics is further highlighted by the lamina and petiole hyponastic responses to the different FR intensities creating the different R:FR ratios (Fig. 4). If data collection would have occurred only at the start of the experiment (T = 0) and after 24 h (T = 24), the conclusions would have been far less subtle. Based on T0 and T24 time points alone, it could be concluded that a dosage with an R:FR = 0.4 and higher is not sufficient to elicit a hyponastic response, only an elongation response (Fig. 4, A, B, and D). However, we showed that all R:FR reductions led to an initial hyponastic response, yet the mild FR treatments converge to WL control conditions 24 h after the start of treatment. Also, here the initial responses could be very sensitive to even mild FR increases, whereas longer-term consolidation and acceleration of the responses involve mechanisms that require stronger FR fluence rates to be activated. Secondly, the synchronous rapid oscillations of both petiole and lamina angles during FRw treatment were not previously described. These oscillations were seemingly not induced during local supplemental FR treatment at the leaf tip, nor were they visible in WL controls. Interestingly, even though *pif4pif5pif7* no longer responded to supplemental FR with leaf elevation, the fast oscillations in FRw were still induced, albeit less pronounced (Fig. 6, G to I). Measuring leaf movements at a lower time resolution in image acquisition, such as in previously published setups, could not have detected these rapid modulations specific to FRw enrichment. It is so far unknown what is the nature of these rapid modulations specific to FRw exposure, and follow-up studies may resolve if these involve specific regulation, are related to biomechanics, and/or are the product of combined acceleration of both leaf elongation and leaf upward movement.

Another interesting observation is that the upward movement of the petiole was preceded by the elongation of the petiole (Fig. 3I, Supplementary Fig. S1B). This difference in timing is consistent with previous observations made on much younger plants under different day/night regimes (Dornbusch et al. 2014), suggesting that, at least in Arabidopsis, FR robustly promotes petiole elongation first, followed by upward movement. However, the opposite order has been described for marsh dock (*Rumex palustris*), a species know to escape flooding using a combination of petiole elongation and hyponasty. Here, petiole elongation will only occur after a petiole angle threshold has been reached via petiole hyponasty (Cox et al. 2003). Future studies could resolve the molecular underpinnings of the differential timing of petiole elongation versus hyponasty in Arabidopsis and other species.

### Insights from spatial resolution

Next to the temporal resolution that permitted close monitoring of the kinetics of plant movement, the resolution in leaf organ tracing enabled us to distinguish nuanced differences between petiole and lamina responses. Under WL conditions, petiole and lamina angle changes and elongation followed a very similar pattern (Fig. 3). The different FR treatments and *pif* mutants, either or not in combination with different light intensities, show that petiole and lamina sometimes respond differently (Figs. 4 to 6). Combining various background PAR levels and locations of perception of low R:FR treatment further highlighted response differences between lamina and petiole angles (Fig. 5). Both local FR and FRw elicited a lamina hyponastic response in the 2 lower background light treatments (PAR = 40 and PAR = 80). This was different for the petiole hyponastic response at those 2 lower PARs, where unlike for FRw, local FR on the leaf tip did elicit a response (Fig. 5, A and D, Supplementary Fig. S3).

Previous research has shown that an auxin flow from the leaf tip to the petiole base is required and sufficient to cause a hyponastic petiole response (Pantazopoulou et al. 2017; Küpers et al. 2023). It is possible that this leaf tip-induced flow results in a stronger auxin gradient in the petiole compared with FRw, where putative local auxin synthesis on both abaxial and adaxial sides of the petiole itself could counterbalance a gradient, yet promote petiole elongation. Previous work by Millenaar et al. (2009) showed the involvement of auxin in low PAR-induced hyponasty. If we assume that low PAR itself also creates an abaxial−adaxial auxin gradient to promote hyponasty (Fig. 5, A, D, and G), then perhaps the additional effect of FR tip-derived auxin is relatively less, thus creating only mild additional hyponasty.

Additionally, angle responses to FRw exposure revealed a difference in response timing between the lamina and the petiole, with the lamina reaching the highest point at the beginning of the night versus the end of the night for the petiole (Fig. 3, A and B). The differences in responses between the petiole and the lamina were further highlighted when exposing the plants to different FRw dosages (Fig. 4). Although both petiole and lamina required the same FRw dosage to maintain their elevated angle after 24 h, the initial lamina angle increase was the highest in the strongest FR treatments, therefore showing a gradual response to the dosage of FRw during the first half of the treatment (Fig. 4B). The petiole hyponastic response on the other hand was identical between all 4 supplemental FR treatments during the first 10 h of treatment. Moreover, *pif7 and pif4pif5* showed a difference in junction angle not only with Col-0 during FRw treatment but also between the 2 mutants (Fig. 6, B, E, and H), indicating that the lamina–petiole junction serves as an additional pivot point in leaf elevation and positioning control that can be specifically controlled.

The differences in lamina and petiole responses for both elongation and angle could indicate a difference in either molecular regulation or timing of this regulation for (differential) elongation between the lamina and the petiole. We found that laminas responded faster to FR treatment than did petioles. Plus, where the lamina maintained its hyponastic response, regardless of background fluence rates, the petiole exhibited an interaction between background fluence rates and the location of FR perception. Thirdly, *pif* mutant combinations displayed varying relative impact to the petiole–lamina junction angles. The faster response for the lamina could hypothetically be explained by the auxin flow from leaf tip to petiole base that is initiated by FR light (Küpers et al. 2023). This flow will naturally first pass through the lamina, before it reaches the petiole where the differential growth response is driven from its basalmost zone (Küpers et al. 2023). Although the differential elongation for laminas is not described, this would likely happen at the lamina–petiole junction. This junction does indeed seem to be specially regulated, since different *pif* mutants have different relative effects on junction versus lamina and petiole angles. Future studies might resolve whether the velocity of the auxin flow or the size of the differential elongation zone is related to the difference in timing between the lamina and petiole angle responses, focusing on the petiole bases and petiole–lamina junction to set up abaxial–adaxial cell growth gradients versus the lamina tip as the likely source of auxin.

## Conclusion

Taken together, we present a low-cost and modular imaging setup and analysis pipeline that provides high temporal resolution for both plant movement and growth. Using this setup and including several light treatment comparisons and combinations, we have discussed details of tissue-specific responses and the importance of insights into the kinetics to correctly interpret differential leaf elongation responses.

## Materials and methods

### Plant materials and growth conditions

Arabidopsis (*Arabidopsis thaliana*) Col-0, *pif7*, *pif4pif5*, and *pif4pif5pif7* (Pantazopoulou et al. 2017) seeds were stratified at 4 °C in the dark for 3 to 6 d on Primasta soil and transplanted to individual 70 mL Primasta-filled pots after ∼9 d in the light. Plants were kept in WL short-day conditions (9 h light, 15 h dark, 70% relative humidity, 20 °C) for 28 d. Light conditions consisted of ∼140 *µ*mol m^−2^ s^−1^ PAR (400 to 700 nm), resulting in an estimated phytochrome photostationary state (PSS) value of 0.79 and an R:FR ratio of 1.5 (Supplementary Fig. S4A) as the default control conditions (Valoya BX-120 modules, NS1 + FR). Plants of 28 d were selected for experiments when the petiole of their 5th youngest leaf was between 4.5 and 5.5 mm long. This leaf was used in all experiments as the focal leaf. Surrounding leaves that blocked view of the camera on the 5th youngest leaf were removed to facilitate downstream automated image analyses.

### Experimental light and treatment conditions

Plants were grown at 140 *µ*mol m^−2^ s^−1^ PAR and transferred to their respective experimental condition at 28 d. Local FR enrichment was performed using individual L735–06 AU LEDs (USHIO) with a peak emission at 735 nm, reducing the R:FR ratio to ∼0.1 at the sites of application, which was the leaf tip. FRw enrichment was achieved with supplemental light from FR-only BX-120 LED bars (Valoya). PAR variations were achieved by adjusting the background light intensity to the desired condition. The treatments consisted of either a range of R:FR ratios, from 0.1 to 0.6, in 140 *µ*mol m^−2^ s^−1^ PAR, or an R:FR ratio of 0.1 under different PAR levels: 40, 80, 140, and 180 *µ*mol m^−2^ s^−1^ PAR conditions, respectively (Supplementary Fig. S4).

### Image acquisition setup

**Figure 7.**
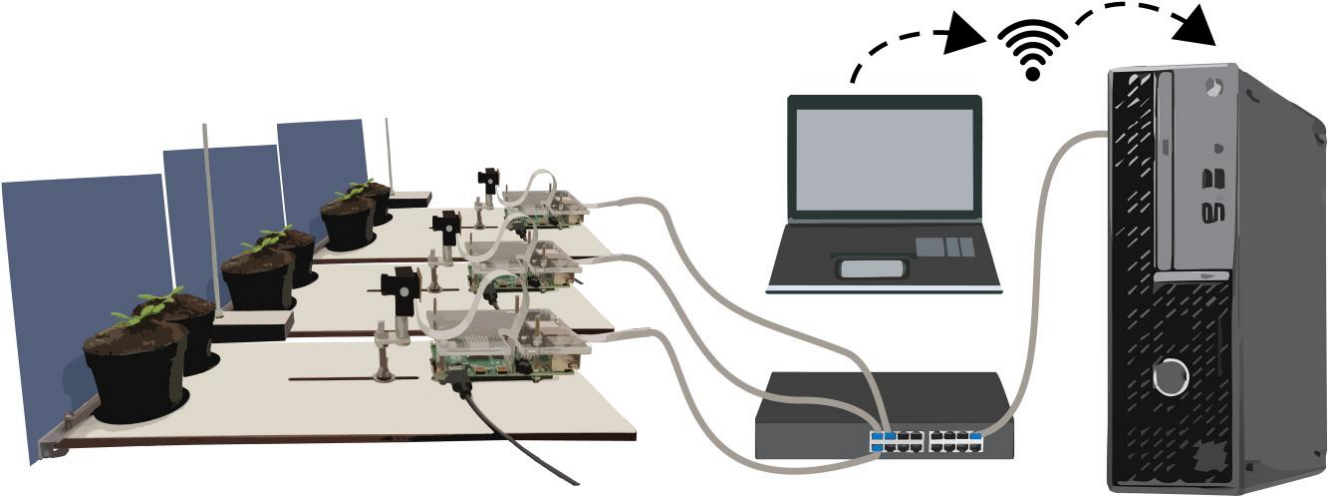
Imaging setup for leaf angle measurements. Two plants in single pots are placed in front of the RPi-controlled camera. Ten of these RPi and camera combinations are connected to a desktop in a private network via a switch. The desktop can be accessed directly or via a remote desktop connection.

Image acquisition was done using 10 RPi 4 model B's (Raspberry Pi Foundation) and 10 RPi Cameras model F (Waveshare Electronics) that support IR imaging, shown in Fig. 7. We custom-built a Pi mounting plate, and 2 pots were placed in fixed positions at 15 cm distance of the cameras. The setup was fitted with a blue resopal background to facilitate downstream image analyses. Three IFR LEDs TSUS540 (Vishay Semiconductors) mounted on a transparent bar above the plants were used per plant for imaging during the night with a peak emission wavelength at 950 nm. Images were taken every 60 s for 24 h and initially stored on a 64 GB micro SD card (SanDisk). Access and control of the RPi's was achieved with a separate computer using a switch (-D-LINK), a wired private network for the Pi's via NAT32 IP Router (NAT Software), and VNC viewer (RealVNC). Data transfer from the RPi's to the separate computer was achieved via open-source software Syncthing (Syncthing Foundation). Time keeping for the RPi's was achieved using Meinberg Network Time Keeping (NTP) package (Meinberg Funkuhren GmbH & Co KG).

### Image analyses and leaf measurements

Because 2 plants are imaged per 1 camera, pictures were cropped using IrfanView 4.58 to result in 1 plant per image and to reduce computational time in downstream analyses. Cropped images were further analyzed in a 2-step Python pipeline using 2 purpose-built scripts, based on OpenCV and ran in Spyder 5.1.5 using Python 3.9.12 The full, open-source scripts with descriptions per step are accessible at https://github.com/Pierik-Lab. The first step consists of defining the starting points for the tracking program. Rosette center, petiole–lamina junction, and leaf tip were defined with a locator in the first image and in the last image (rosette center). Secondly, a region of known length is selected for downstream conversion from pixels to mm. This information was stored per leaf and used as basis for the second step of the python pipeline. The second python script performed the actual image analyses and user-defined point position tracking. Images were split in R, G, and B channels to separate the nighttime pictures from the light pictures and to enhance the CRST tracking. The *x* and *y* coordinates of each point of interest were defined per subsequent frame using OpenCV based on information inside and outside the bounding box of 40 × 60 pixels surrounding the user-defined points via an adaptive discriminative correlation filter (Bradski 2000; Lukežič et al. 2018; Xu et al. 2019). A CSV file per plant was generated containing the *x* and *y* coordinates of the points of interest in pixels per frame. Further downstream analyses, angle calculations, and graph creation were performed using Rstudio 2021.09.1+372 and R 4.1.2 based on the *x* and *y* coordinates and are accessible at https://github.com/Pierik-Lab. Petiole, lamina, leaf, and junction angles were retrieved via triangulation between meristem, petiole–lamina junction, and the leaf tip. The data processing pipeline in R was made such that all data per plant was retained separately and could be exported grouped in a CSV file. Single plants within an experiment were time synced based on the day–night transition in the pictures. Relative angles and elongation data was obtained by subtraction of the average of the first 5 images from each subsequent image.

### Statistical analyses

Because a datapoint was generated every minute, a grouping of data was done every 2 h for statistical testing. One or 2-way ANOVAs were performed with R base functions and package emmeans (Lenth 2022), combined with Tukey tests. Script is available at https://github.com/Pierik-Lab.

### Accession numbers

This article contains no new sequence data.

## Supplementary Material

kiae097_Supplementary_Data

## Data Availability

The data underlying this article will be shared on reasonable request to the corresponding author.

## Supplementary data

The following materials are available in the online version of this article.

**Supplementary Figure S1.** Plant response kinetics to WL or WL with supplemented FR light.

**Supplementary Figure S2.** Extended timeframe plant response kinetics with WL or WL and supplemental local FRt.

**Supplementary Figure S3.** Plant response kinetics and spectral composition of reduced intensity FRt treatments.

**Supplementary Figure S4.** Spectral composition of the different light treatments.

## Dive Curated Terms

The following phenotypic, genotypic, and functional terms are of significance to the work described in this paper:

PIF4 Gramene: AT2G43010
PIF4 Araport: AT2G43010

PIF7 Gramene: AT5G61270
PIF7 Araport: AT5G61270

petiole AmiGo: PO:0020038

lamina AmiGo: PO:0025060

PIF5 Gramene: At3g59060
PIF5 Araport: At3g59060

leaf AmiGo: PO:0025034

leaf angle Planteome: TO:0000206
