# Supplementary material for: A low-cost open-source imaging platform reveals spatiotemporal insight into leaf elongation and movement
Source: Plant Physiol. 2024 Feb 24;195(3):1866–79. doi: 10.1093/plphys/kiae097 (PMC11213255; doi:10.1093/plphys/kiae097)
Supplement: kiae097_Supplementary_Data [file kiae097_supplementary_data.zip › kiae097_Supplementary_Data.pdf]

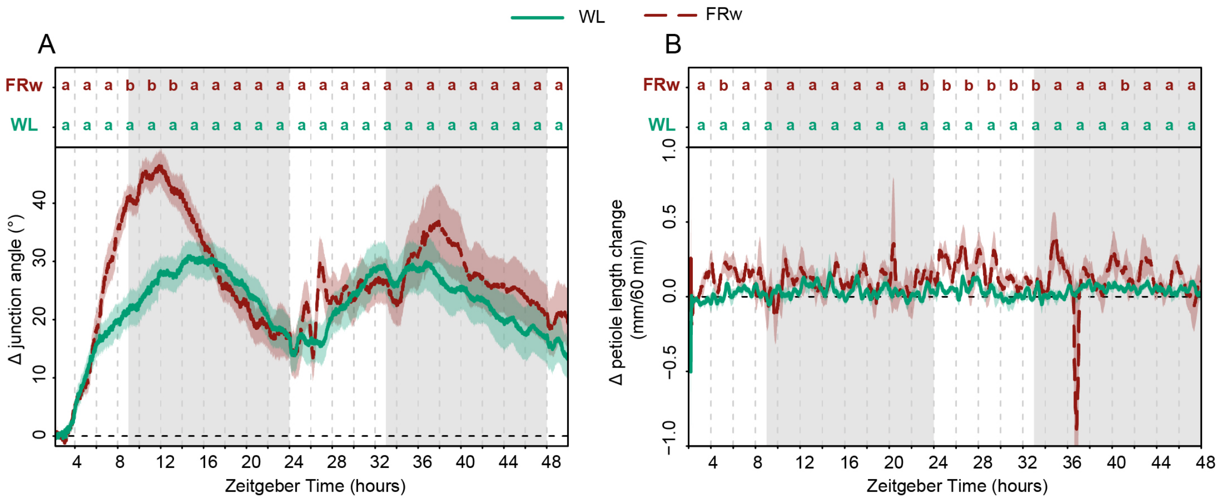

Supplemental Figure S1. Plant response kinetics to white light (WL) or WL with supplemented far-red (FR) light. Relative angle change for the lamina-petiole junction (A), and speed of petiole elongation over 60 minute periods (B). Grey areas indicate night without light or FR exposure. Plants were followed for 48 hours, treated with whole plant supplemental FR (FRw), R:FR = 0.1, or control white light (WL) with PAR = 140 for both treatments. Treatment start time at ZT=2, WL n=7, FRw n= 6. Letters indicate $p < 0.05$, calculated per every 2 hours using one-way ANOVA and Tukey post-hoc test.

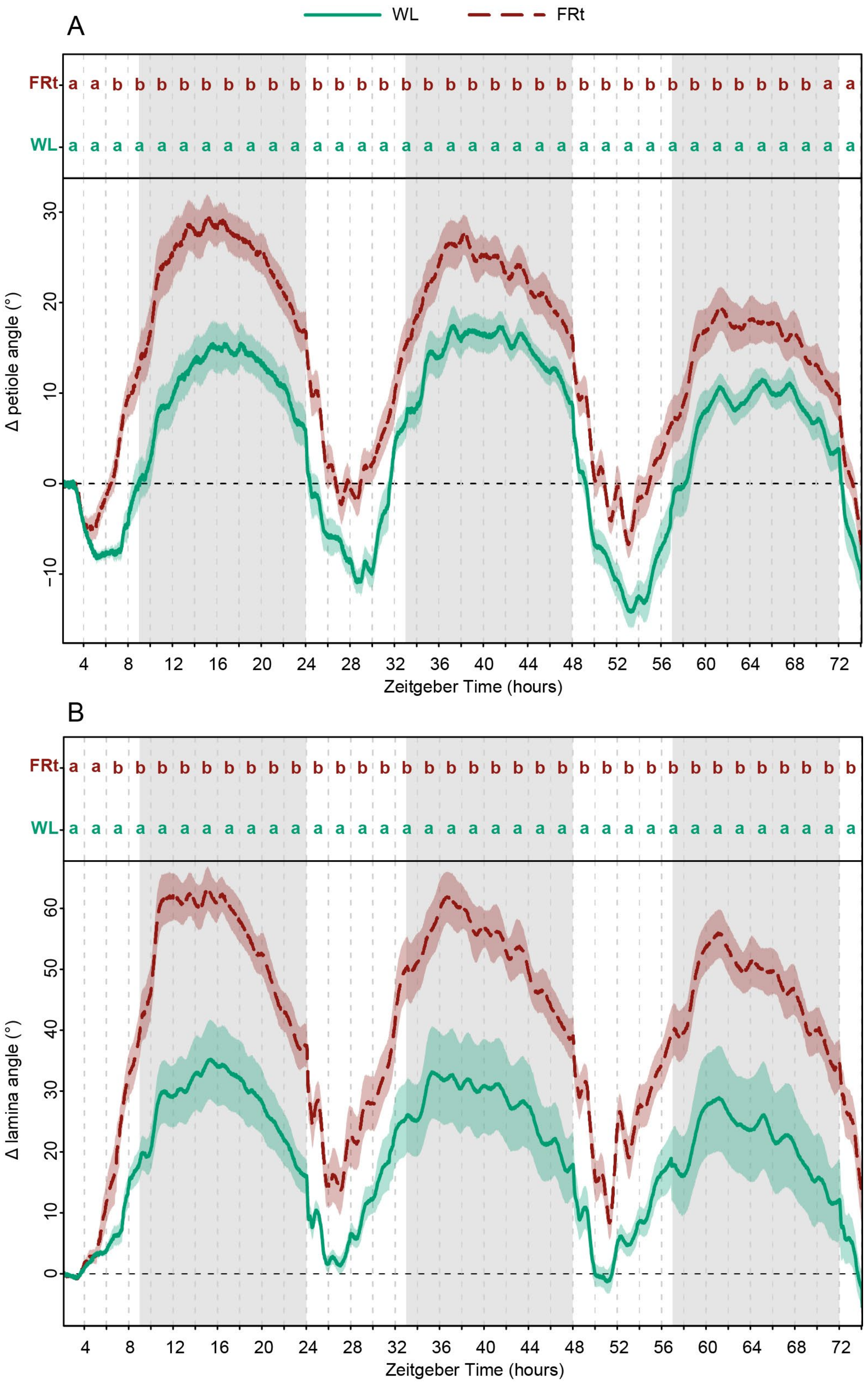

WL
FRt
A
FRt
WL
Δ petiole angle (°)
Zeitgeber Time (hours)
B
FRt
WL
Δ lamina angle (°)
Zeitgeber Time (hours)

Supplemental Figure S2. Extended timeframe plant response kinetics with white light (WL) or WL and supplemental local far red at the leaf tip (FRt). Differential petiole angle (A) and lamina angle (B) over 72 hours are shown for WL and local FR treatment at the leaf tip (FRt) with R:FR = 0.1, and PAR = 140 for both treatments. Grey areas indicate night without light or FR exposure. Treatment start time at ZT=2, WL n=7, FRt n= 8. Letters indicate p < 0.05, calculated per every 2 hours using one-way ANOVA and Tukey post-hoc test.

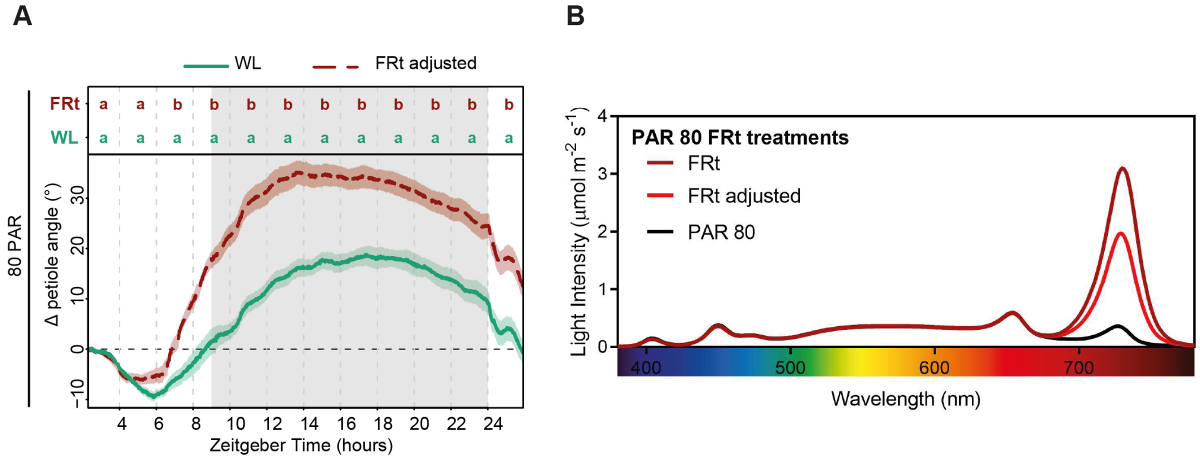

Supplemental Figure S3. Plant response kinetics and spectral composition of reduced intensity FRt treatments. (A) Plant response kinetics for reduced supplemental far-red light at the the leaf tip (FRt) intensity at 80 µmol m^{-2} s^{-1} photosynthetically active radiation (PAR). Grey areas indicate night without light or FR exposure. Treatment start time at ZT=2, WL n=8, FRt n= 10. Letters indicate p < 0.05, calculated per every 2 hours using one-way ANOVA and Tukey post-hoc test. (B) Spectral composition of FRt treatments. Background light WL was reduced with approximately one third from 140 to 80 PAR, shown as PAR 80. Original FRt fluence rate of the LEDs, shown as FRt, was similarly adjusted for this experiment, shown as FRt adjusted.

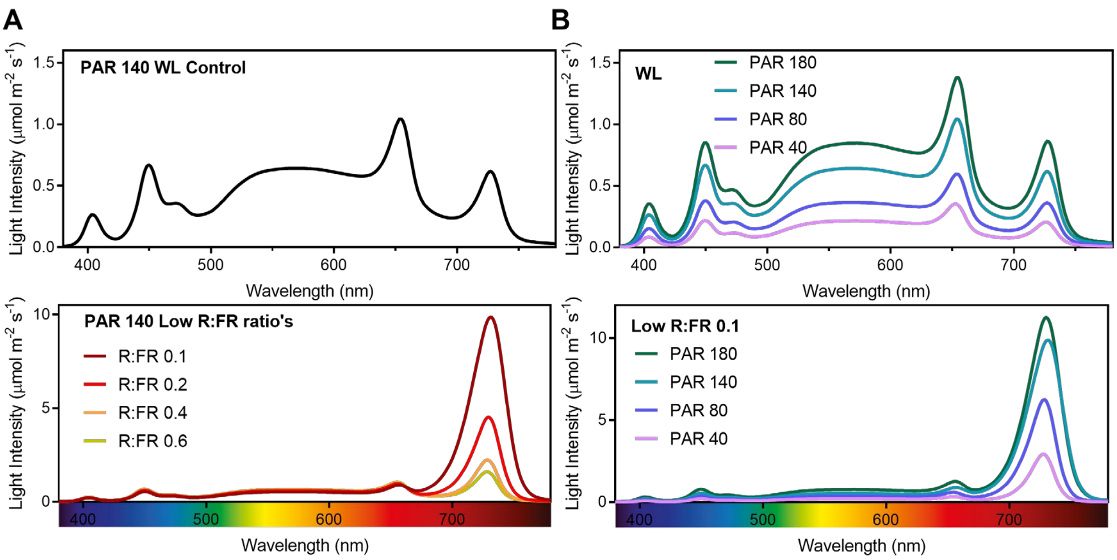

Supplemental Figure S4. Spectral composition of the different light treatments. Control condition of 140 PAR, with R:FR= 1.5 for the upper panel, and the different FR treatments in the lower panel (A). At the upper panel different WL spectra with R:FR=1.5, together with the corresponding Low R:FR ratios in the lower panel (B). R, red; FR, far red; WL, white light; PAR, photosynthetically active radiation.
