# Supplementary material for: A low-cost open-source imaging platform reveals spatiotemporal insight into leaf elongation and movement
Source: Plant Physiol. 2024 Feb 24;195(3):1866–79. doi: 10.1093/plphys/kiae097 (PMC11213255; doi:10.1093/plphys/kiae097)
Supplement: kiae097_Supplementary_Data [file pp.01330.2023-s03.pdf › pp.01330.2023-s03.pdf]

Plant Physiology®

A low-cost and open-source imaging platform reveals spatiotemporal insight into Arabidopsis leaf elongation and movement

Lisa Oskam, Basten Snoek, Chrysoula Pantazopoulou, Hans van Veen, Sanne Matton, Rens Dijkhuizen, and Ronald Pierik

Review Timeline:

Submission Date:	30-Aug-2023
Editorial Decision:	25-Sep-2023
Revision Received:	08-Jan-2024
Accepted:	25-Jan-2024

25-Sep-2023

Prof. Dr. Ronald Pierik
Wageningen University & Research
Laboratory of Molecular Biology
Wageningen 6708 PB
Netherlands

RE: A low-cost and open-source imaging platform reveals spatiotemporal insight into Arabidopsis leaf elongation and movement

Dear Dr. Pierik:

We have received reviews of your manuscript, which you may view at the end of this letter. On the basis of the advice received and my own evaluation, I believe that, pending revision, this article may ultimately be acceptable for publication in Plant Physiology. Here are the 3 major points I would like you to address

- make sure all the information required to make such a system is available (including if 3D printing was used), see comment of reviewer 1.
- be more explicit about your novel findings (some of the data confirms what was previously reported)
- consider showing data for some mutants as either a validation or even better uncovering something new
- please make sure to return a point-by-point answer to the reviewers comments

When you are ready to submit your revision, click the below link and find the red arrow under your Author Tasks heading for this revision submission.

If you want to visit this version of the manuscript, you may click the link below and then click on the folder that reads "Post Decision Manuscripts".

Link Not Available

When uploading the revised version of this article, please be sure to include as complete an answer as possible to the referees' criticisms during the first round of reviews in the "response to reviewers" section.

Plant Physiology considers a revision for up to 60 days after a manuscript has been provisionally accepted; limited extension of this timeframe is at the discretion of the Monitoring Editor. A reminder notice will be sent to you 2 weeks before the deadline, at which time you may choose to contact the editorial office to request an extension; if so, please provide a justification for your request. After the expiration of this deadline, the article would need to be submitted as a resubmission and would be handled as such.

Thank you very much for giving us an opportunity to review this work. I look forward to receiving the next version.

Sincerely,

Plant Physiology Board of Editors

Want to add this revision deadline to your calendar? Click below!

--- Reviewer comments:
Reviewer #1 (Comments for the Author):

The manuscript titled "A low-cost open-source imaging platform reveals spatiotemporal insight into Arabidopsis leaf elongation and movement" is a methods paper describing detailed tracking of leaf movement in both light and dark conditions. Using their imaging and semi-automated analysis pipeline, petiole and leaf tip can be independently tracked relative to the rosette center to

independently calculate the petiole, leaf lamina, and whole leaf's angles and length. Their pipeline was tested by comparing white light conditions to low R:Fr ratio conditions. Under low R:Fr, the lamina and leaf angle peak before the petiole angle, and petiole length but not lamina length increases significantly. Increasing FR fluence rates also generally increases petiole and lamina responses. Localized FR treatment of the leaf tip induces hyponastic responses via increased petiole angle under all light intensities, while FR induced hyponastic responses are only seen with whole plant FR treatment at high light intensities.

Major comments
It is mentioned nine times that this method can elucidate molecular mechanisms, but this manuscript focuses on the observations of leaf responses to environmental conditions. No mutants were used, or any attempt to delve into molecular mechanisms was presented. While I can see the potential to use this method to tease apart function using genetic, chemical, and molecular techniques, I recommend being conservative on this point in the text unless there is additional experimental evidence provided that mutants in shade avoidance syndrome genes can be distinguished from wild type by their varied or altered hyponastic dynamics using this system, or further insight into an established molecular mechanism was determined

Please provide the part numbers for the assembled system from the sources when possible, and if any parts were 3D printed (Raspberry Pi cases and camera mounting systems) please provide the STL files or upload to a public repository (github repository associated with this paper or hosted on Thingiverse.com) to facilitate the replication of your system.

Minor comments
1. It is unclear which of the observations in response to the environment are novel. It is mentioned that a few phenotypes corroborate previous results, but it may be worth pointing out cases where measured responses to R:FR changes had not previously been reported.
2. Figure 2 legend: image labels do not correspond to the text description.
3. Lines 182 and 185: "Figure 33G" and "Figure 33H" should be relabeled as Figure 3
4. Figure 3: statistic formatting on graphs is not consistent.
5. Line 443: The exact distance has been left out
6. Supplemental Figure 1B y-axis label: "lenght" change to length
7. Supplemental Figure 3: What is the fluence rate of FR? What is the R:FR ratio?
8. Methods, line 443: "We custom-built a Pi mounting plate from" Should from be form?

Reviewer #2 (Comments for the Author):

In this manuscript, Oskam et al. introduce a cost-effective and modular imaging system capable of tracking the elongation and angle of the petiole, lamina, and entire leaf. They elegantly demonstrate how this setup can effectively monitor the dynamics of leaf movements and growth in response to low R/FR light conditions, allowing for the identification of spatial separation in the hyponastic response of the petiole and lamina. The authors discovered that these responses are influenced by the specific light conditions to which the plant is exposed. In the future, employing their setup, it will be intriguing to investigate how light signaling and circadian mutants regulate the growth and movement of the petiole and lamina in an organ-specific manner.

The authors collected an extensive amount of high-quality and valuable data. Their open-source semi-automated image analysis pipeline is well-documented and can be implemented in any lab with some basic knowledge of Python and R.
The manuscript is well-written, and the question addressed is clearly outlined. The results strongly support the conclusions. Below are a few points that may help to improve the manuscript:

Minor points:
- To demonstrate the sensitivity and limitations of the system, one could consider using a mutant with reduced leaf hyponasty, such as the pif mutants.
- Could the authors offer insights or recommendations regarding the potential applications of the setup described in the manuscript for specific plant organs and species? Is it adaptable for investigating leaf movement in species with larger leaves or for monitoring hypocotyl and stem segment elongation?
- In Sup. Fig. 2 It appears that the change in the petiole angle decreased on the third night compared to the first two nights. If this is indeed the case, it raises several intriguing questions:
1. Does the lamina angle also change differently after three days?
2. Is this phenomenon exclusive to FRt or also observable in FRw?
3. How long can the imaging setup continue to track changes in leaf angle?
- To enhance the clarity of Figure 1, consider including a cartoon that illustrates the various parts of the leaf.
- The letters in the Fig. 2 legend do not match those in the Figure.
- Line 185 typo "Figure 33H"
- Line 255 needs to be FRt instead of FRtip

Dear editor and reviewers,

Thank you very much for your helpful comments on our manuscript. Below is a point-by-point explanation of how we used your feedback to improve our manuscript.

Editor:

We have received reviews of your manuscript, which you may view at the end of this letter. On the basis of the advice received and my own evaluation, I believe that, pending revision, this article may ultimately be acceptable for publication in Plant Physiology. Here are the 3 major points I would like you to address

- make sure all the information required to make such a system is available (including if 3D printing was used), see comment of reviewer 1.

All information is provided, and no 3D printing was used. For additional clarity, we added that the IR LEDs were mounted on a transparent bar above the plants.

- be more explicit about your novel findings (some of the data confirms what was previously reported)

We added clarifications in several points of the manuscript to highlight the novel findings.

- consider showing data for some mutants as either a validation or even better uncovering something new

We have now added data from a number of new experiments investigating the roles of PIF transcription factor, using pif7, pif4pif5 *and* pif4pif5pif7 *mutants, to the pape. This is shown in new figure 6. These data show that the procedures can detect important roles of these transcription factors in regulating leaf movement, consistent with previous publications. Interestingly, these canonical shade avoidance regulators displayed a difference in hyponastic regulation between the petiole and the lamina, further highlighting the lamina petiole junction as an additional, and largely understudied, point of leaf positioning.*

- please make sure to return a point-by-point answer to the reviewers comments

Please find the point-by-point answers to the reviewers below.

Reviewer #1 (Comments for the Author):

The manuscript titled "A low-cost open-source imaging platform reveals spatiotemporal insight into Arabidopsis leaf elongation and movement" is a methods paper describing detailed tracking of leaf movement in both light and dark conditions. Using their imaging and semi-automated analysis pipeline, petiole and leaf tip can be independently tracked relative to the rosette centre to

independently calculate the petiole, leaf lamina, and whole leaf's angles and length. Their pipeline was tested by comparing white light conditions to low R:Fr ratio conditions. Under low R:Fr, the lamina and leaf angle peak before the petiole angle, and petiole length but not lamina length increases significantly. Increasing FR fluence rates also generally increases petiole and lamina responses. Localized FR treatment of the leaf tip induces hyponastic responses via increased petiole angle under all light intensities, while FR induced hyponastic responses are only seen with whole plant FR treatment at high light intensities.

Major comments

It is mentioned nine times that this method can elucidate molecular mechanisms, but this manuscript focuses on the observations of leaf responses to environmental conditions. No mutants were used, or any attempt to delve into molecular mechanisms was presented. While I can see the potential to use this method to tease apart function using genetic, chemical, and molecular techniques, I recommend being conservative on this point in the text unless there is additional experimental evidence provided that mutants in shade avoidance syndrome genes can be distinguished from wild type by their varied or altered hyponastic dynamics using this system, or further insight into an established molecular mechanism was determined

Thank you for pointing this out. We have followed both suggestions: we made some textual revisions as requested, and we we added data from a number of new experiments investigating the roles of PIF transcription factors, using pif7, pif4pif5 *and* pif4pif5pif7 *mutants, to the paper. This is shown in new figure 6. These data show that the procedures can detect important roles of these transcription factors in regulating leaf movement, consistent with previous publications. Interestingly, these canonical shade avoidance regulators displayed a difference in hyponasty regulation between the petiole and the lamina, further highlighting the lamina-petiole junction as an additional, and largely understudied, point of leaf positioning.*

Please provide the part numbers for the assembled system from the sources when possible, and if any parts were 3D printed (Raspberry Pi cases and camera mounting systems) please provide the STL files or upload to a public repository (github repository associated with this paper or hosted on Thingiverse.com) to facilitate the replication of your system.

No parts were 3D printed for this set-up. For additional clarity, we added that the IR LEDs were mounted on a transparent bar above the plants.

Minor comments

1. It is unclear which of the observations in response to the environment are novel. It is mentioned that a few phenotypes corroborate previous results, but it may be worth pointing out cases where measured responses to R:FR changes had not previously been reported.

We added the requested clarifications in the text.

2. Figure 2 legend: image labels do not correspond to the text description.

Labels adjusted

3. Lines 182 and 185: "Figure 33G" and "Figure 33H" should be relabelled as Figure 3

Relabelled

4. Figure 3: statistic formatting on graphs is not consistent.

We have adjusted the formatting

5. Line 443: The exact distance has been left out

Distance has been added (15 cm)

6. Supplemental Figure 1B y-axis label: "lenght" change to length

We removed the spelling error

7. Supplemental Figure 3: What is the fluence rate of FR? What is the R:FR ratio?

Thanks for noting this. We have added panel B to supplemental figure 3, showing the original spectrum of the FRt treatment in Figure 5 in the main text, and the adjusted FRt treatment for Supplemental Figure 3.

8. Methods, line 443: "We custom-built a Pi mounting plate from" Should from be form?

Removed the unnecessary word

Reviewer #2 (Comments for the Author):

In this manuscript, Oskam et al. introduce a cost-effective and modular imaging system capable of tracking the elongation and angle of the petiole, lamina, and entire leaf. They elegantly demonstrate how this setup can effectively monitor the dynamics of leaf movements and growth in response to low R/FR light conditions, allowing for the identification of spatial separation in the hyponastic response of the petiole and lamina. The authors discovered that these responses are influenced by the specific light conditions to which the plant is exposed. In the future, employing their setup, it will be intriguing to investigate how light signalling and circadian mutants regulate the growth and movement of the petiole and lamina in an organ-specific manner.

The authors collected an extensive amount of high-quality and valuable data. Their open-source semi-automated image analysis pipeline is well-documented and can be implemented in any lab with some basic knowledge of Python and R.
The manuscript is well-written, and the question addressed is clearly outlined. The results strongly support the conclusions.
Below are a few points that may help to improve the manuscript:

Minor points:
To demonstrate the sensitivity and limitations of the system, one could consider using a mutant with reduced leaf hyponasty, such as the pif mutants.

As suggested by the reviewer, we added pif mutants pif7, pif45 *and* pif457 *in a sixth figure to the manuscript.*

Could the authors offer insights or recommendations regarding the potential applications of the setup described in the manuscript for specific plant organs and species? Is it adaptable for investigating leaf movement in species with larger leaves or for monitoring hypocotyl and stem segment elongation?

Because the system is based on point selection in the pictures through a separate software pipeline that is fully available as open source script, other points could also be followed through time. This might require a smaller or larger bounding box for point selection and adjustment of thresholds in the first script when the image is split in different channels. Additionally, other camera's with different focal distances are available from the same manufacturer, allowing pictures from a larger distance. This would aid in capturing larger plants and leaves. The angle under which the pictures are made is also adjustable, because the points are user selected. The main requirements are a high time resolution, to allow the software to look for the point in the subsequential picture.

We have slightly elaborated this section in the Dicussion in the first paragraph under the heading "Spatial and temporal resolution from adjustable image acquisition and analysis pipeline".

In Sup. Fig. 2 It appears that the change in the petiole angle decreased on the third night compared to the first two nights. If this is indeed the case, it raises several intriguing questions:
1. Does the lamina angle also change differently after three days?

The lamina angle follows a similar pattern, and is now added to the supplemental figures as Supplemental figure 2B

2. Is this phenomenon exclusive to FRt or also observable in FRw?

This phenomenon is also observable in FRw, as was also previously reported by Michaud et al. (2017).

3. How long can the imaging setup continue to track changes in leaf angle?

The imaging set-up could in principle track changes in leaf angle until the memory card is full, ~3 Gb per day, with a ~50 Gb of free space means approximately 16 days. If the pictures are stored externally in a cloud storage, there is no technical limit. Additional obstacles could be growing out of frame, or newly developed leaves growing and blocking the camera view of course.

To enhance the clarity of Figure 1, consider including a cartoon that illustrates the various parts of the leaf.

Thank you, this is a very helpful suggestion. We added a new panel to Figure 1 containing a picture that illustrates the various parts of the leaf

The letters in the Fig. 2 legend do not match those in the Figure.

Letters adjusted

Line 185 typo "Figure 33H"

Typo adjusted

Line 255 needs to be FRt instead of FRtip

Adjusted in the text

25-Jan-2024
Prof. Dr. Ronald Pierik
Wageningen University & Research
Laboratory of Molecular Biology
Wageningen 6708 PB
Netherlands

MSID: PP2023-RA-01330R1
MS TITLE: A low-cost and open-source imaging platform reveals spatiotemporal insight into Arabidopsis leaf elongation and movement

Dear Dr. Ronald Pierik:

I am pleased to inform you that your revised manuscript "A low-cost and open-source imaging platform reveals spatiotemporal insight into Arabidopsis leaf elongation and movement" has been accepted for publication in Plant Physiology. Your manuscript will be checked for consistency with journal formatting and image standards by one of our Science Editors, who will contact you shortly. They may have a number of queries and suggestions for you. Please keep in mind that they work to increase the online visibility of your article and its attraction for readers from the widest of backgrounds. You will have the opportunity to review any revisions, and I hope you will work constructively with them to get the most out of your research. You will also have the opportunity to review all changes when you receive the author proofs for your article.

Once validated for publication, your article will be sent to a Science Editor for a final review of your manuscript and source files. After your final files are uploaded, your article will appear in the next available issue and the online Preview version of your article will be posted after your signed Copyright and Checklist forms and final source files are received.

Please be sure to include the following in your manuscript file:
Harvard style citations
List of all author contributions and funding information at the beginning of manuscript after the titles and author list.
List all supplemental materials associated with the article, with titles, at the end of the ms, before the references and acknowledgments section.
Complete Figure Legends for all figures appearing in the article listed before the Literature Cited.

Thank you and congratulations on your Plant Physiology paper!

Sincerely,
Plant Physiology Board of Editors

=====
FIRST AUTHOR PROFILES
=====

We recognize the hard work that goes into being First Author of a Plant Physiology paper by publishing a "First Author Feature" for each First Author alongside your paper.

If the first author(s) would like to submit information for a feature on Facebook and Twitter, fill out the form by clicking on the link below:

Plant Physiology First Author Feature Form: https://forms.office.com/r/ZBCGZfhm8F
If you cannot access the form through the link above, please contact Rachel at rbelsky@aspb.org.

To upload an image of yourself to accompany your profile, please put your photo in the correct journal folder at the link below. You must label your image: Author First Name Last Name Article Title.

First Author Profile Image Dropbox: https://www.dropbox.com/scl/fo/xz029p7qjr2jml2rp2kmq/h?dl=0&rlkey=6fkxcaqmxx35ap152b2osy1fz

=====
IMPORTANT REMINDER: PEER REVIEW REPORTS
=====

If you opted to publish a peer review report along with your article during the original submission process, it will be prepared by the editorial staff and publicly posted with your manuscript, inside the zip file that contains any other supplemental material. As a reminder, the peer review report is a public record of all comments from editors and reviewers, as well as your prior responses, as you received them in the decision letters for each draft of your manuscript. If you agreed to publish this report and have changed your mind, or are not sure if you selected this option, please contact the editorial office as soon as possible before signing the license agreement from our publisher.

-- Reviewer comments:
Reviewer #1 (Comments for the Author):

They have addressed my prior concerns through new experiments and editing. However, in the new sections, Col0 is used when Col-0 is the standard for this ecotype.

Reviewer #2 (Comments for the Author):

All of my comments and suggestions were addressed.
